# Widespread ground motion distribution caused by rupture directivity during the 2015 Gorkha, Nepal earthquake

**DOI:** 10.1038/srep28536

**Published:** 2016-06-23

**Authors:** Kazuki Koketsu, Hiroe Miyake, Yujia Guo, Hiroaki Kobayashi, Tetsu Masuda, Srinagesh Davuluri, Mukunda Bhattarai, Lok Bijaya Adhikari, Soma Nath Sapkota

**Affiliations:** 1 Earthquake Research Institute, University of Tokyo, 1-1-1 Yayoi, Bunkyo-ku, Tokyo, Japan; 2 National Geophysical Research Institute, Uppal Road, Hyderabad, India; 3 Department of Mines and Geology, Lainchour, Kathmandu, Nepal

## Abstract

The ground motion and damage caused by the 2015 Gorkha, Nepal earthquake can be characterized by their widespread distributions to the east. Evidence from strong ground motions, regional acceleration duration, and teleseismic waveforms indicate that rupture directivity contributed significantly to these distributions. This phenomenon has been thought to occur only if a strike-slip or dip-slip rupture propagates to a site in the along-strike or updip direction, respectively. However, even though the earthquake was a dip-slip faulting event and its source fault strike was nearly eastward, evidence for rupture directivity is found in the eastward direction. Here, we explore the reasons for this apparent inconsistency by performing a joint source inversion of seismic and geodetic datasets, and conducting ground motion simulations. The results indicate that the earthquake occurred on the underthrusting Indian lithosphere, with a low dip angle, and that the fault rupture propagated in the along-strike direction at a velocity just slightly below the *S*-wave velocity. This low dip angle and fast rupture velocity produced rupture directivity in the along-strike direction, which caused widespread ground motion distribution and significant damage extending far eastwards, from central Nepal to Mount Everest.

The Gorkha earthquake occurred on 25 April 2015 (UT) in the north part of central Nepal, causing widespread damage with more than 8,000 fatalities. In the Himalayan region, including Nepal, the Indian plate is colliding with the southern margin of the Eurasian plate, and the Indian lithosphere underthrusts beneath the Himalayas^1^ along the Main Himalayan Thrust (MHT), which reaches the ground surface at the Main Frontal Thrust (MFT; Fig. 1a). This underthrusting generates large Himalayan earthquakes, the hazards of which have been noted for decades, together with the seismic vulnerability of the countries around the Himalayas^2^,^3^. According to the tectonics described above and the result of the Global CMT Project (GCMT)^4^, the focal mechanism of the Gorkha earthquake was dip-slip rupture with a strike of west-northwest (WNW).

Rupture directivity is a combined effect of rupture propagation, the earthquake source radiation pattern, and particle motion polarization on seismic ground motions^5^. This effect is known to cause directional variations in seismic ground motion and damage^6^,^7^,^8^,^9^, and to occur if a strike-slip or dip-slip rupture propagates to a site in the along-strike or updip direction, respectively^10^. However, although the focal mechanism of the 2015 Gorkha earthquake was dip-slip faulting, as mentioned above, rupture directivity was found in the Kathmandu Valley, which is located in the nearly along-strike direction.

The ground motions observed by the Department of Mines and Geology (DMG) of Nepal in Kathmandu during the earthquake (upper traces in Fig. 1b)^11^ show large pulse-like waveforms, especially in the vertical component, although the later parts of the horizontal components were complicated by the basin effects of the Kathmandu Valley. Such ground motion pulses are considered to be firm evidence of rupture directivity^6^,^7^,^8^,^9^. The occurrence of rupture directivity was also confirmed by the regional acceleration seismograms^12^ in the lower traces in Fig. 1b, where the strong-motion duration in the forward direction is shorter than in the backward direction^10^. The teleseismic displacement seismograms in Fig. 2 show both the large pulse-like waveforms and shorter ground motion duration in the forward direction, as shown in ref. 13.

Here, we first explore the reasons why along-strike rupture directivity occurred during the dip-slip Gorka earthquake, by performing a joint source inversion of waveform and geodetic datasets. We next examine the relationship between enhanced ground motion amplitudes and rupture directivity by conducting ground motion simulations.

## Results

### Joint source inversion

**Figure 1:**
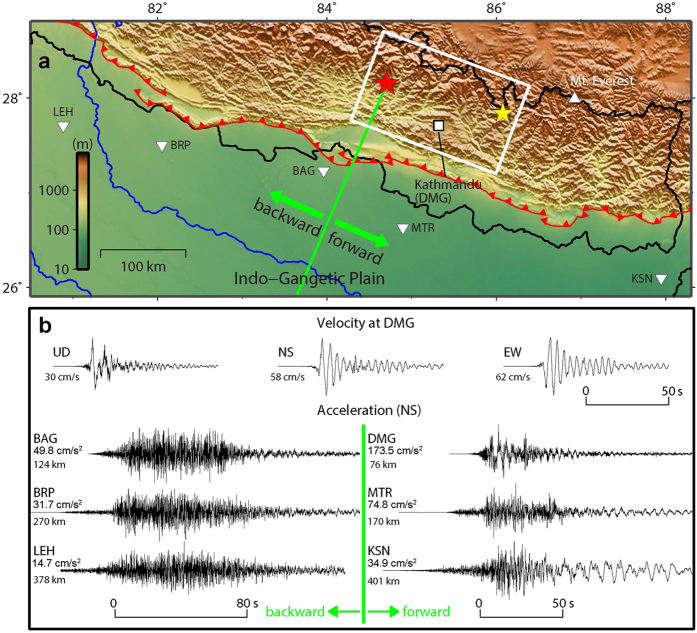
Index map and seismograms in Nepal and India for Gorkha earthquake. (**a**) Hypocentres of the main shock and largest aftershock are indicated by red and yellow stars. We located the source fault of the earthquake (white rectangle) based on aftershock data and the quick GCMT solution. Red curves with triangles represent the MFT. (**b**) Ground velocities observed by DMG^11^ in Kathmandu during the main shock (top), and ground accelerations observed in northern India along the MFT, compiled by NGRI^12^ (lower). Accelerations in forward and backward directions of fault rupture propagation were plotted in lower right and left halves, respectively. The map was generated using Generic Mapping Tools^40^ 4 (http://gmt.soest.hawaii.edu/).

In order to explore the reasons underlying the apparent inconsistency mentioned above, it is crucial to investigate the rupture process of the Gorkha earthquake. First, we constructed the source fault model of strike = 290° and dip = 7° (Fig. 1a), using the distribution of the main shock and aftershocks, and the quick GCMT solution. It is noted here that the dip angle of the source fault is as low as 7°. We then carried out a joint inversion of waveform and geodetic datasets (see Methods). Two types of waveform datasets were available for this inversion: 1) the global seismograms shown in Supplementary Fig. 1a, which were obtained from the Global Seismographic Network through the Data Management Center of the Incorporated Research Institutions for Seismology, and 2) the local seismograms shown in Supplementary Fig. 1b, which were observed at strong motion stations^14^ and high-rate GPS stations^15^. Two types of geodetic datasets were also available for this inversion: 1) horizontal and vertical ground deformations at static GPS stations^15^ shown in Supplementary Fig. 2a,b and 2) line-of-sight ground deformations shown in Supplementary Fig. 2c, which were derived from the processed InSAR image^16^.

The resultant total slip distribution from the inversion is shown in Fig. 3a, with a maximum value of 6.4 m. The calculated seismic moment was 8.6 × 10^20^ Nm, which yielded an *M*_*w*_ of 7.9. The fit of the synthetics to the observations was also shown in Supplementary Figs 1 and 2. Most of the synthetics show good fit, but those for the horizontal components of local seismograms underestimate the observations because of the limitations of the 1-D velocity structure constructed (see Methods). Snapshots of the slip distribution were taken every 10 s after the rupture initiation at the hypocentre (Fig. 3b), showing that the rupture propagated eastward nearly along the strike, at an almost constant velocity of about 3.3 km/s, which is slightly lower than the *S*-wave velocity of 3.5 km/s on the source fault.

### Rupture directivity for dip-slip faulting

As with the schematic illustration in Supplementary Fig. 3 for a strike-slip earthquake such as the 1995 Kobe earthquake^17^, in this case along-strike rupture propagation caused the directivity effects, producing constructive interference of seismic waves in the forward direction. *S*-waves from the fault segments arrived almost simultaneously along the rupture direction. They resulted in the pulse-like shape^6^,^10^ and long-period feature^18^ of the strong motion seismograms such as those observed in the Kathmandu Valley^11^ (Fig. 1b), and a zone of large ground motion spreading beyond the main rupture area. The latter feature cannot be generated by factors other than rupture directivity.

However, for dip-slip earthquakes, rupture directivity has not been thought to occur during along-strike rupture propagation, such as in the Gorkha earthquake. Actually, if the rupture velocity is close to the S-wave velocity and the faulting mechanism is nearly uniform, constructive interference of seismic waves or a ground motion pulse can occur in any rupture direction following the schematic mechanism shown in Supplementary Fig. 3, but a ‘large’ ground motion pulse has to occur for the identification of rupture directivity. This condition can be satisfied if large ground motions are generated along the rupture direction. For a typical dip slip with a dip angle of 45°, large ground motions are generated only along the updip direction because of its *S*-wave radiation pattern, and therefore, the rupture directivity is visible only during the updip rupture propagation of a typical dip-slip earthquake.

**Figure 2:**
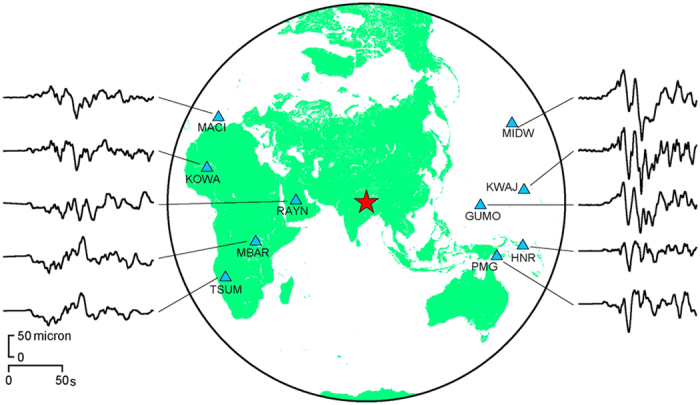
Teleseismic displacement seismograms for Gorkha earthquake. Vertical components of seismograms in forward and backward directions of fault rupture propagation from the hypocentre (red star) are plotted on right and left sides, respectively, of the map of stations (blue triangles). They were filtered with a passband of 0.005 to 0.4 Hz. The map was generated using Generic Mapping Tools^40^ 4 (http://gmt.soest.hawaii.edu/).

In contrast, the rupture directivity cannot be seen during along-strike rupture propagation of a typical dip-slip earthquake, as shown in Fig. 4, because the nodal plane of the *S*-wave radiation pattern extends in the along-strike direction. However, if the dip angle is as low as that of the Gorkha earthquake, the ground above the dip slip is located in a lobe of the radiation pattern, and the rupture directivity occurs during along-strike rupture propagation (Fig. 5). The strong motion seismograms observed in the Kathmandu Valley (Fig. 1b and Supplementary Fig. 1b) and regional and teleseismic waveforms (Figs 1b and 2) provide, for the first time, conclusive evidence of rupture directivity during the along-strike rupture propagation of a low-angle dip-slip earthquake.

### Ground motion distribution

In ref. 19, nearly 4,000 macroseismic effects of the Gorkha earthquake had been collected, and converted into shaking intensities through detailed assessments. The distribution of resultant intensities^19^ in Fig. 6a shows that intensities of 7 or larger are mostly concentrated in a 100 km wide zone extending east-southeast from the main shock epicentre. However, since no intensity was obtained between the longitudes of 86 and 87°E along the extension, we cannot determine the eastern end of the high intensity zone.

To compensate for these missing data, we calculated the distribution of the fatality rate, which is the ratio of the number of fatalities to the total population in a district (see Methods). According to this distribution, shown in Fig. 7, we found districts between 86 and 87°E to have fatality rates of 0.01 to 0.1%, which correspond to an intensity of 7 (ref. 20). In these far eastern districts, included is the district of Mount Everest, where avalanches induced by seismic ground motions killed 20 people and injured 120 people^21^. Therefore, it has been realized that the high intensity zone was extended from the main shock epicentre in central Nepal to Mount Everest. Enhanced shaking due to along-strike rupture directivity of the Gorkha earthquake likely played an important contributing role to this widespread ground motion distribution.

To confirm the above, we conducted ground motion simulations using the finite-element method (FEM) with a voxel mesh^22^ (see Methods). A preliminary model of three-dimensional (3-D) velocity structure had been constructed for this simulation, based on a geological profile in central Nepal^23^, global relief data^24^, a global model of Earth’s crust^25^, and a geological model of the Kathmandu Valley^26^ (inset of Fig. 6b). Simulated ground motions were filtered with a passband of 0.05 to 0.4 Hz (see Methods), which covers significant frequency contents of observed velocity seismograms^14^, but the buildings that collapsed and caused fatalities would likely be most sensitive to higher frequencies.

It was found that the resultant distribution of peak ground velocities in Fig. 6b simulates the intensity distribution (Fig. 6a) augmented by the fatality rate distribution (Fig. 7) fairly well, if we refer to the relationship of intensities and peak ground velocities^27^. The fatality rate was used only to compensate for the missing part of the intensity distribution. In particular, large ground velocities are spread far to the east in a similar manner to the augmented intensity distribution. However, moderate ground velocities also extend south-east to the Indo-Gangetic Plain beyond the MFT. This is, in part, consistent with the observation that at least 78 people were killed and 560 were injured in India^21^, although the intensities in the southernmost part of Nepal beyond the MFT were limited, as shown in Fig. 6a.

To clarify the contribution of the basin effect to the directivity-basin coupling^28^, which generated the larger ground motion pulses in the basin called Kathmandu Valley, we constructed another velocity structure model (hereafter ‘modified velocity structure’) by setting the sediment velocities in the basin to be equal to the basement velocities, and then performed a ground motion simulation using the modified velocity structure. In Fig. 6c, the ground velocities and their Fourier spectra from this simulation are compared with those from the previous simulation, at a site of sediments 750 m thick in the basin. The comparison indicates that the horizontal ground velocities were amplified twice or more by the sediments, while no amplification was found in the vertical component. In particular, at resonant frequencies of 4 to 5 s, the horizontal velocity spectra were amplified by as much as ten times.

## Discussion

In addition to the rupture directivity studied above, the ‘fling step’ effect has been proposed as another contributor of long-period ground motion pulses^29^,^30^ and it was also identified in the ground motion pulses of the Gorkha earthquake^14^. Since, in this effect, slip dislocation is assumed to directly ‘fling’ the near-fault ground^30^, the ground motion pulse produced by this effect should include the near-field term of the analytical solution^31^ (ref. 30). However, the near-field term decays rapidly for long distances in inverse proportion to the distance squared^29^. Therefore, although the ground motion pulses in the Kathmandu Valley could be characterized by a combination of the rupture directivity and fling step effects, rupture directivity is a key factor causing the large ground motion spread far to the east during the Gorkha earthquake. This is also confirmed by the regional acceleration duration in Fig. 1b and the teleseismic waveforms in Fig. 2.

**Figure 3:**
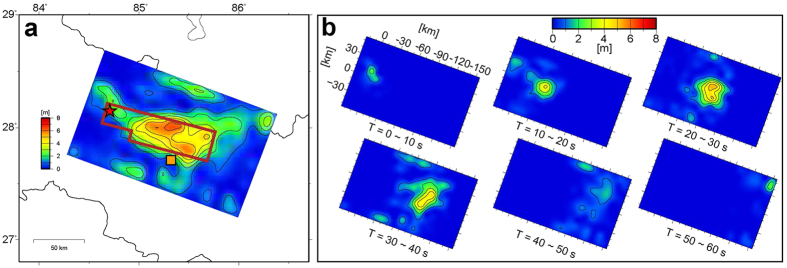
Results of the source inversion. (**a**) Distribution of resultant total slips. The main part of the distribution is outlined by a brown line. The red star and orange square indicate the hypocentre and Kathmandu, respectively. (**b**) Snapshots of slip distribution every 10 s, illustrating nearly constant rupture propagation eastwards. The map was generated using Generic Mapping Tools^40^ 4 (http://gmt.soest.hawaii.edu/).

To compare the rupture directivity and fling step contributions to ground motions in the near field of the Gorkha earthquake, we calculated ground motions using the far- and intermediate-field terms, or only the near-field term of the analytical solution in an infinite medium^31^. The line source in Fig. 5 was modified with a length of 60 km, a seismic moment of 4.5 × 10^20^ Nm (*M*_*w*_ 7.7), and a rupture velocity of 3.1 km/s, to fit to the zone of largest slips in the north of Kathmandu (Fig. 3a). The results of this calculation in Fig. 8 show that, even in the near field, the rupture directivity effect is mostly larger than the fling step effect. However, in the waveforms in Kathmandu, the north–south component of the fling step effect is comparable to that of the rupture directivity effect. Therefore, in the north–south ground velocities observed by DMG (Fig. 1b), the first southward pulse arriving earlier was due to the fling step effect, because the effect includes contributions before the *S*-wave arrival. The second northward pulse corresponds to the rupture directivity effect. It is also noted from Fig. 8 that the shape of a fling step pulse is mainly controlled by constructive interference like a rupture directivity pulse.

## Methods

### Schematic directivity illustration

**Figure 4:**
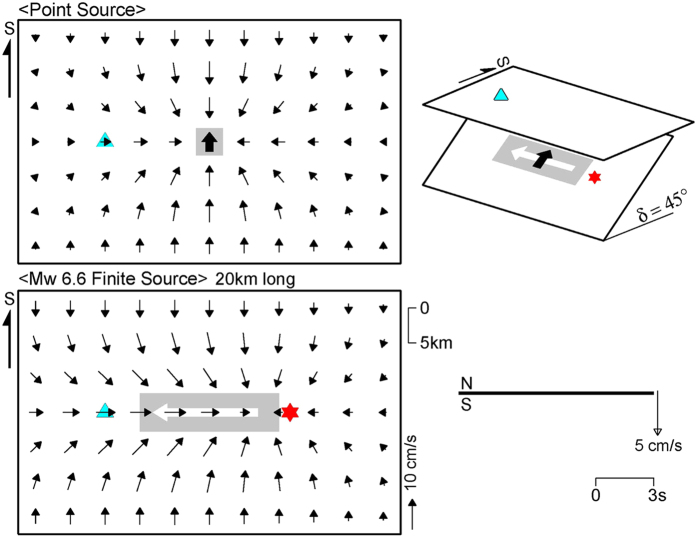
Schematic illustration of rupture directivity for a typical dip-slip earthquake (45° dip). Configuration is shown in upper right diagram. The upper and lower left diagrams show ground motion patterns of point and finite source models (grey zones), respectively. Thick arrows in black and white indicate directions of slip and rupture, respectively. The red star is the rupture initiation point, and the sky blue triangle denotes a station. The lower right diagram shows the north–south component of ground motion at the station, which vanishes because of the nodal plane.

**Figure 5:**
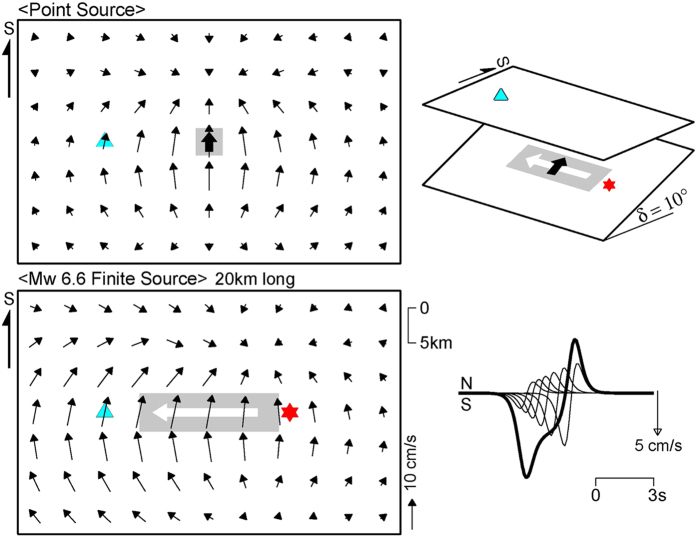
Schematic illustration of rupture directivity for a low-angle dip-slip earthquake (10° dip). Configuration is shown in upper right diagram. The upper and lower left diagrams show ground motion patterns of point and finite source models, respectively. Thick arrows in black and white indicate directions of slip and rupture, respectively. The red star is the rupture initiation point, and the sky blue triangle denotes a station. In the lower right diagram, the thick curve represents constructive interference of north–south ground motions from fault segments (thin curves), illustrating the mechanism of rupture directivity pulse from a low-angle dip-slip finite source.

**Figure 6:**
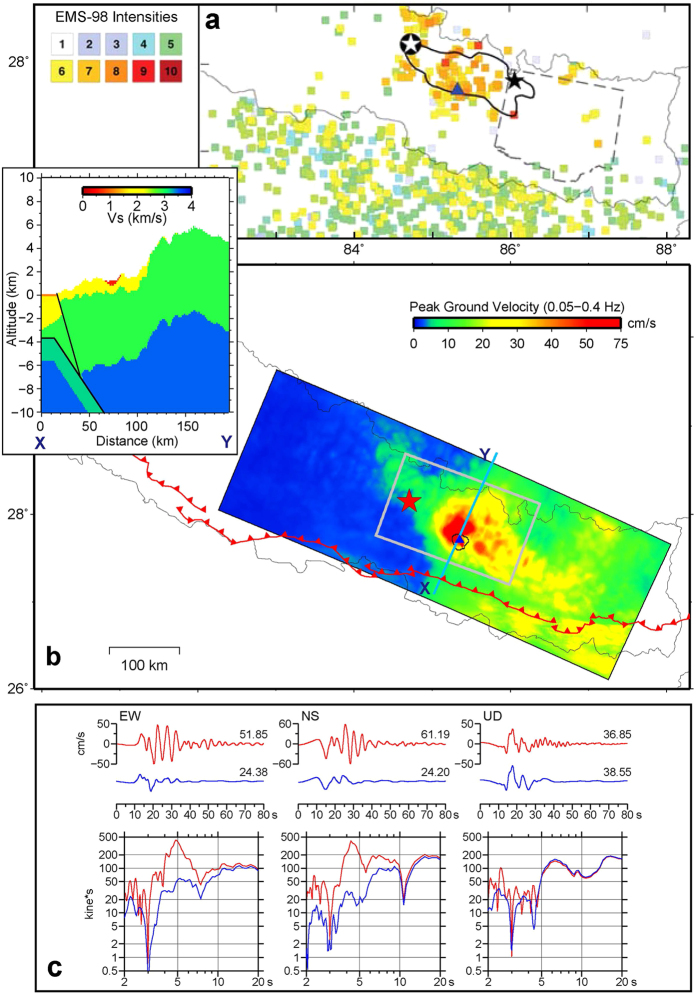
Distributions of observed intensities^19^ and simulated ground velocities. (**a**) The Intensity distribution for the Gorkha earthquake. The white and black stars represent the main shock and largest aftershock, respectively. The blue triangle is the location of Kathmandu. The approximate rupture areas of the 2015 Gorkha and 1934 Bihar-Nepal earthquakes are outlined by solid black and dashed grey lines, respectively^19^ (© Seismological Society of America). (**b**) Maximum ground velocities simulated with voxel FEM. Red curves with triangles represent the MFT. The red star, grey rectangle, and blue triangle are the hypocentre, source fault, and Kathmandu, respectively. The inset shows the assumed velocity structure along profile X–Y. (**c**) Ground velocities simulated with the assumed velocity structure (upper red traces) or the modified velocity structure (upper blue traces). Their Fourier spectra are also shown in the lower half. The map was generated using Generic Mapping Tools^40^ 4 (http://gmt.soest.hawaii.edu/).

**Figure 7:**
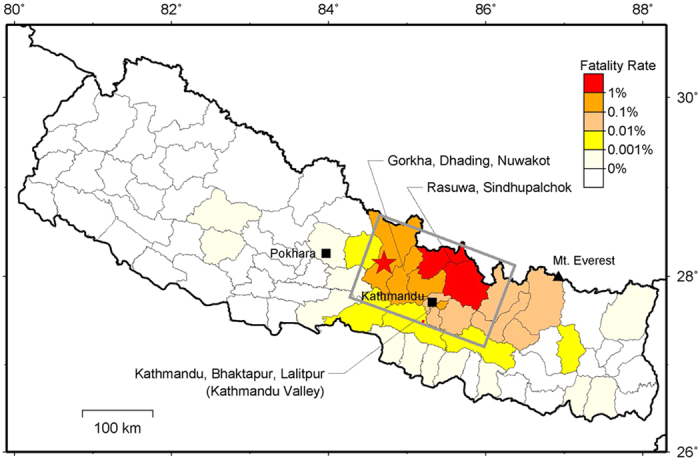
Distributions of fatality rates in all districts of Nepal. The area in each district is coloured according to fatality rate: red (greater than 1%), orange (0.1 to 1%), light orange (0.01 to 0.1%), yellow (0.001 to 0.01%), light yellow (smaller than 0.001%), or white (0%). The names of districts with higher rates are displayed. The grey rectangle and red star denote the source fault and hypocentre, respectively. The map was generated using Generic Mapping Tools^40^ 4 (http://gmt.soest.hawaii.edu/).

**Figure 8:**
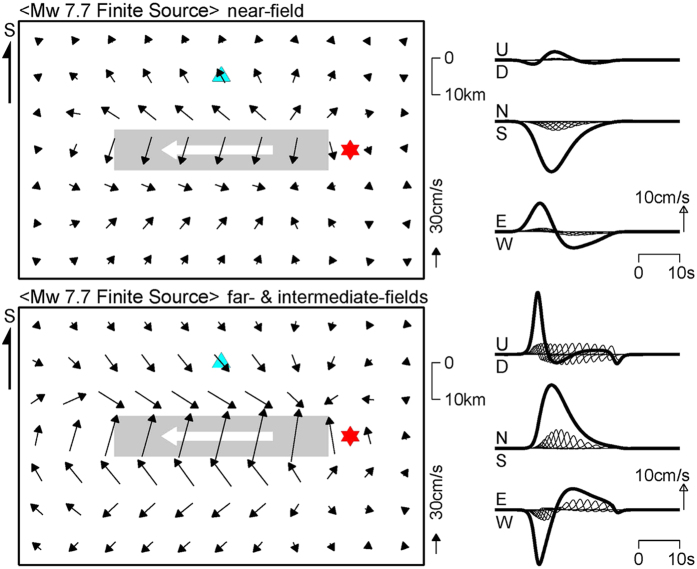
Ground motion patterns in the near field of the Gorkha earthquake and waveforms at Kathmandu. A line source 60 km long is assumed with a seismic moment of 4.5 × 10^20^ Nm (*M*_*w*_ 7.7). The upper pattern and waveforms are due to fling step pulses produced by the near-field term. The lower pattern and waveforms correspond to rupture directivity pulses produced by the far- and intermediate-terms of the analytical solution^31^. The thick arrow in white indicates direction of rupture. The red star is rupture initiation point, and sky blue triangle denotes Kathmandu. The thick curves in waveforms represent total ground motions consisting of those from fault segments, shown by thin curves.

An infinite medium was assumed, with an *S*-wave velocity (*V*_*S*_) of 3.5 km/s, and a buried point or line source. Ground motions were calculated on the ground above the source, using the intermediate- and far-field *S*-wave terms of the analytical solution in ref. 31. In Figs 4 and 5, dip-slip faulting was assumed along a line source 20 km long with a dip angle of 45° or 10° and a seismic moment of 1.0 × 10^19^ Nm (*M*_*w*_ 6.6). The rupture velocity is 3.15 km/s (90% of *V*_*S*_).

### Source inversion scheme

We performed the source inversion using a least-squares method with smoothing and non-negativity constraints^32^,^33^. The weights of the constraints were determined using the Akaike Bayesian Information Criterion^34^. Green’s functions for teleseismic, strong motion, and geodetic data were computed using 1-D velocity structures derived from CRUST 1.0 (ref. 25), and the methods of refs 35, 36, 37.

### Fatality rate calculation

The fatality rate in each district was calculated from the number of fatalities listed in the press release on 7 May 2015 (ref. 38) divided by the population listed in the 2011 national census^39^. Then, the area of each district was coloured red (greater than 1%), orange (0.1 to 1%), light orange (0.01 to 0.1%), yellow (0.001 to 0.01%), light yellow (smaller than 0.001%), or white (0%). Since the largest aftershock occurred on 12 May 2015, the results do not include its effects.

### Ground motion simulation

We used the FEM, which had been reformulated for a seismic ground motion simulation using voxels (hexahedra or rectangular prisms) in a 3-D mesh with topography and the accuracy of which had been assessed^22^. The 540 × 190 × 64 km mesh for the assumed velocity structure model in this study was configured with voxels of 150 × 150 × 150 m (at depths shallower than 8.4 km) or 300 × 300 × 300 m (otherwise). We conducted ground motion simulations in this velocity structure mesh with the main part of the source model in Fig. 3a, and simulated ground velocities 150 s long were filtered with a passband of 0.05 to 0.4 Hz. This passband was determined based on the limitations of the voxel FEM and velocity structure model.

## Additional Information

**How to cite this article**: Koketsu, K. *et al*. Widespread ground motion distribution caused by rupture directivity during the 2015 Gorkha, Nepal earthquake. *Sci. Rep.* **6**, 28536; doi: 10.1038/srep28536 (2016).

## Supplementary Material

Supplementary Information
