# Author Correction: Widespread ground motion distribution caused by rupture directivity during the 2015 Gorkha, Nepal earthquake

**DOI:** 10.1038/s41598-020-63939-z

**Published:** 2020-04-24

**Authors:** Kazuki Koketsu, Hiroe Miyake, Yujia Guo, Hiroaki Kobayashi, Tetsu Masuda, Srinagesh Davuluri, Mukunda Bhattarai, Lok Bijaya Adhikari, Soma Nath Sapkota

**Affiliations:** 1 Earthquake Research Institute, University of Tokyo, 1-1-1 Yayoi, Bunkyo-ku, Tokyo Japan; 2 National Geophysical Research Institute, Uppal Road, Hyderabad, India; 3 Department of Mines and Geology, Lainchour, Kathmandu Nepal

Correction to: *Scientific Reports* 10.1038/srep28536, published online 23 June 2016

The authors neglected to cite a previous paper showing that the fling effect can appear very close to a very shallow, large slip. This paper is included below as Ref. ^1^. In the original article, this paper should appear as Reference 41 and should be cited as follows. The first paragraph of the Discussion section,

“In addition to the rupture directivity studied above, the ‘fling step’ effect has been proposed as another contributor of long-period ground motion pulses^29,30^ and it was also identified in the ground motion pulses of the Gorkha earthquake^14^. Since, in this effect, slip dislocation is assumed to directly ‘fling’ the near-fault ground^30^, the ground motion pulse produced by this effect should include the near-field term of the analytical solution^31^ (ref. 30). However, the near-field term decays rapidly for long distances in inverse proportion to the distance squared^29^. Therefore, although the ground motion pulses in the Kathmandu Valley could be characterized by a combination of the rupture directivity and fling step effects, rupture directivity is a key factor causing the large ground motion spread far to the east during the Gorkha earthquake. This is also confirmed by the regional acceleration duration in Fig. 1b and the teleseismic waveforms in Fig. 2.”

should read:

“In addition to the rupture directivity studied above, the fling effect^29,30^ has been proposed as another contributor to long-period ground motion pulses at very near distances from a very shallow, large slip^41^. However, as the main part of the Gorkha earthquake was at a depth of 15 km and Kathmandu is located 10 km away from it, this effect would not be perceived there during the earthquake. The near-field term of the analytical solution^31^ can also contribute to near-fault ground motions, but it decays rapidly with distance in inverse proportion to the distance squared^29^. Therefore, although the ground motion pulses in the Kathmandu Valley could be characterized by a combination of the rupture directivity and near-field term effects, rupture directivity is a key factor causing the large ground motion that was spread far to the east during the Gorkha earthquake. This is also confirmed by the duration of regional acceleration demonstrated in Fig. 1b and the teleseismic waveforms demonstrated in Fig. 2.”

In addition, all instances of the phrase “fling step” should be replaced with the phrase “near-field term”.

As a result, the second paragraph of the Discussion section,

“To compare the rupture directivity and fling step contributions to ground motions in the near field of the Gorkha earthquake, we calculated ground motions using the far- and intermediate-field terms, or only the near-field term of the analytical solution in an infinite medium^31^. The line source in Fig. 5 was modified with a length of 60 km, a seismic moment of 4.5 × 10^20^ Nm (*M*_*w*_ 7.7), and a rupture velocity of 3.1 km/s, to fit to the zone of largest slips in the north of Kathmandu (Fig. 3a). The results of this calculation in Fig. 8 show that, even in the near field, the rupture directivity effect is mostly larger than the fling step effect. However, in the waveforms in Kathmandu, the north–south component of the fling step effect is comparable to that of the rupture directivity effect. Therefore, in the north–south ground velocities observed by DMG (Fig. 1b), the first southward pulse arriving earlier was due to the fling step effect, because the effect includes contributions before the *S*-wave arrival. The second northward pulse corresponds to the rupture directivity effect. It is also noted from Fig. 8 that the shape of a fling step pulse is mainly controlled by constructive interference like a rupture directivity pulse.”

should read:

“To compare the rupture directivity and near-field term contributions to ground motions in the near field of the Gorkha earthquake, we calculated ground motions using the far- and intermediate-field terms, or only the near-field term of the analytical solution in an infinite medium^31^. The line source in Fig. 5 was modified with a length of 60 km, a seismic moment of 4.5 × 10^20^ Nm (*M*_*w*_ 7.7), and a rupture velocity of 3.1 km/s, to fit to the zone of largest slips in the north of Kathmandu (Fig. 3a). The results of this calculation in Fig. 8 show that, even in the near field, the rupture directivity effect is mostly larger than the near-field term effect. However, in the waveforms in Kathmandu, the north–south component of the near-field term effect is comparable to that of the rupture directivity effect. Therefore, in the north–south ground velocities observed by DMG (Fig. 1b), the first southward pulse arriving earlier was due to the near-field term effect, because the effect includes contributions before the *S*-wave arrival. The second northward pulse corresponds to the rupture directivity effect. It is also noted from Fig. 8 that the shape of a near-field term pulse is mainly controlled by constructive interference like a rupture directivity pulse.”

In the legend of Figure 8,

“The upper pattern and waveforms are due to fling step pulses produced by the near-field term.”

should read:

“The upper pattern and waveforms are due to the pulses produced by the near-field term.”

These changes affect only the additional Discussion and do not affect any other parts of the Article including the conclusions. The Authors apologise for these changes.
