# Supplementary material for: Widespread ground motion distribution caused by rupture directivity during the 2015 Gorkha, Nepal earthquake
Source: Sci Rep. 2016 Jun 23;6:28536. doi: 10.1038/srep28536 (PMC4917818; doi:10.1038/srep28536)
Supplement: Supplementary Information [file srep28536-s1.pdf]

Supplementary Information

Widespread ground motion distribution caused by rupture directivity during the 2015 Gorkha, Nepal earthquake

Kazuki Koketsu[1,*], Hiroe Miyake[1], Yujia Guo[1], Hiroaki Kobayashi[1], Tetsu Masuda[1], Srinagesh Davuluri[2], Mukunda Bhattarai[3], Lok Bijaya Adhikari[3] & Soma Nath Sapkota[3]

[1] Earthquake Research Institute, University of Tokyo, 1-1-1 Yayoi, Bunkyo-ku, Tokyo, Japan.

[2] National Geophysical Research Institute, Uppal Road, Hyderabad, India.

[3] Department of Mines and Geology, Lainchour, Kathmandu, Nepal.

[*] koketsu@eri.u-tokyo.ac.jp

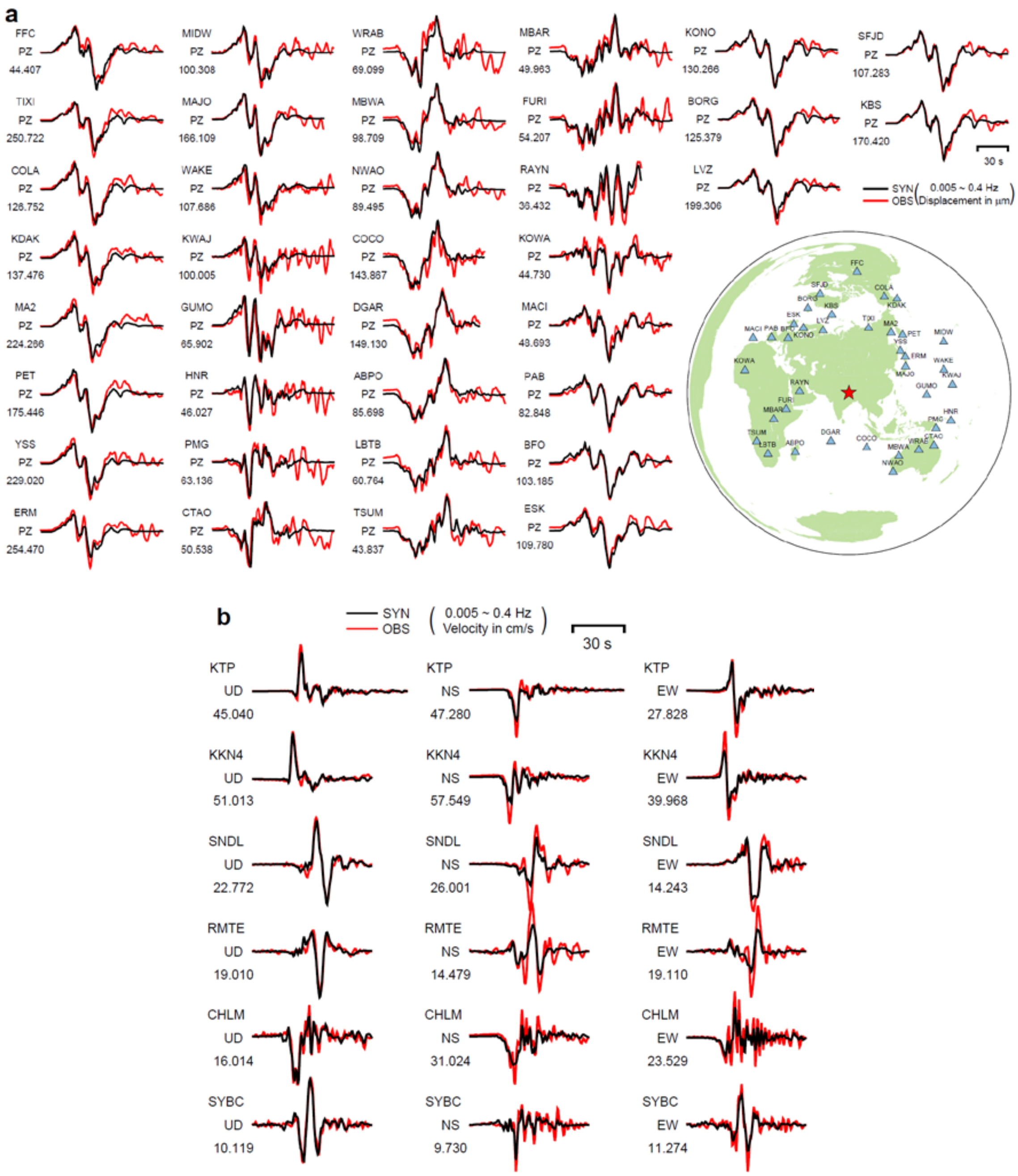

Supplementary Figure 1 | Comparison of observed and synthetic seismograms. a, Seismograms at global teleseismic stations shown in the lower right map. Red star denotes main shock. Peak displacements in μm are written on the left of the seismograms. **b,** Seismograms at strong motion[1] and high-rate GPS[2] stations in Nepal. Peak velocities in cm/s are written on the left of the seismograms. The map was generated using Generic Mapping Tools[5] 4 (http://gmt.soest.hawaii.edu/).

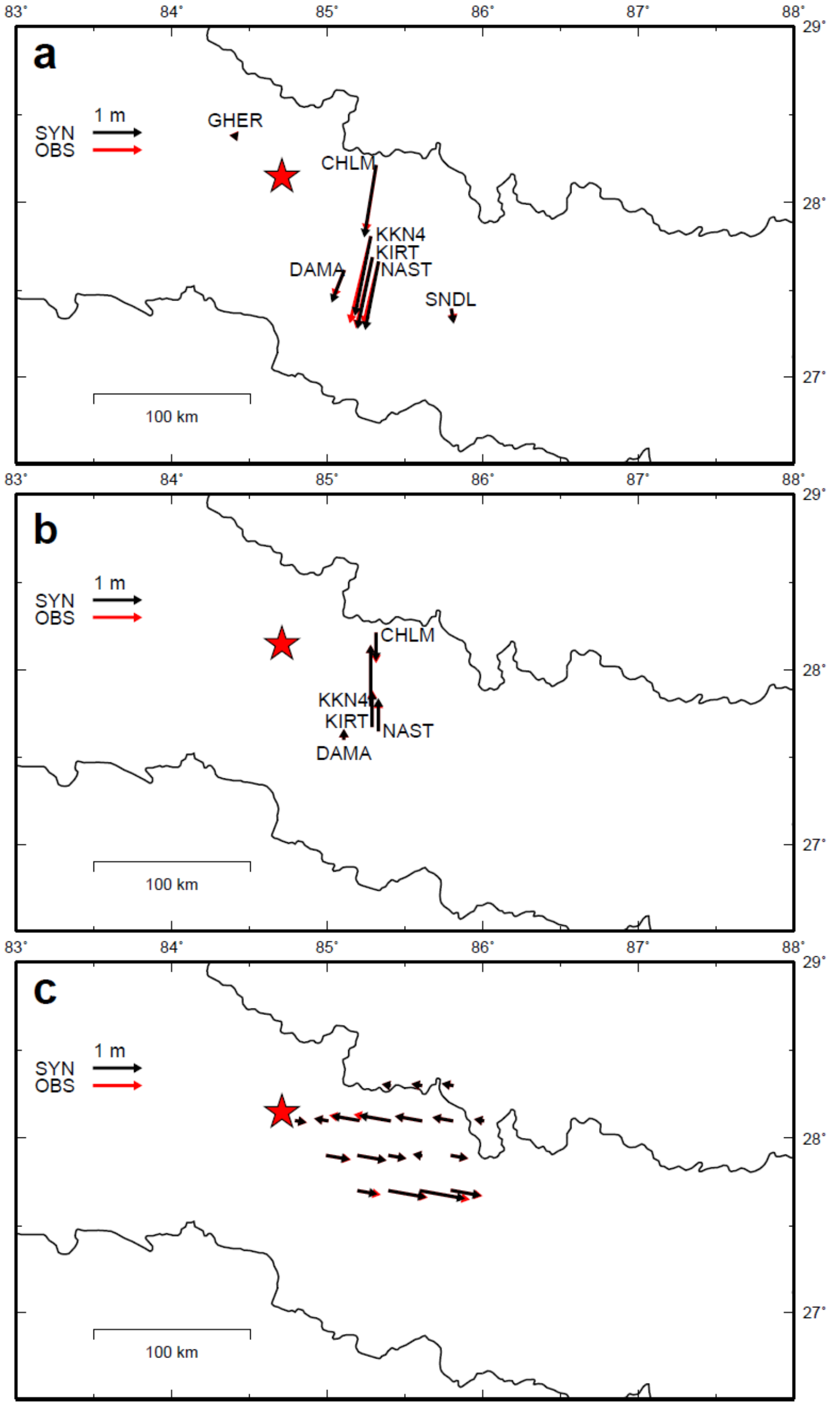

Supplementary Figure 2 | Comparison of observed and synthetic ground deformations. a, Horizontal deformations at seven GPS stations. **b,** Vertical deformations at five GPS stations. **c,** Line-of-sight deformations were derived from the processed InSAR images[3]. Red stars denote the main shock epicentre. The maps were generated using Generic Mapping Tools[5] 4 (http://gmt.soest.hawaii.edu/).

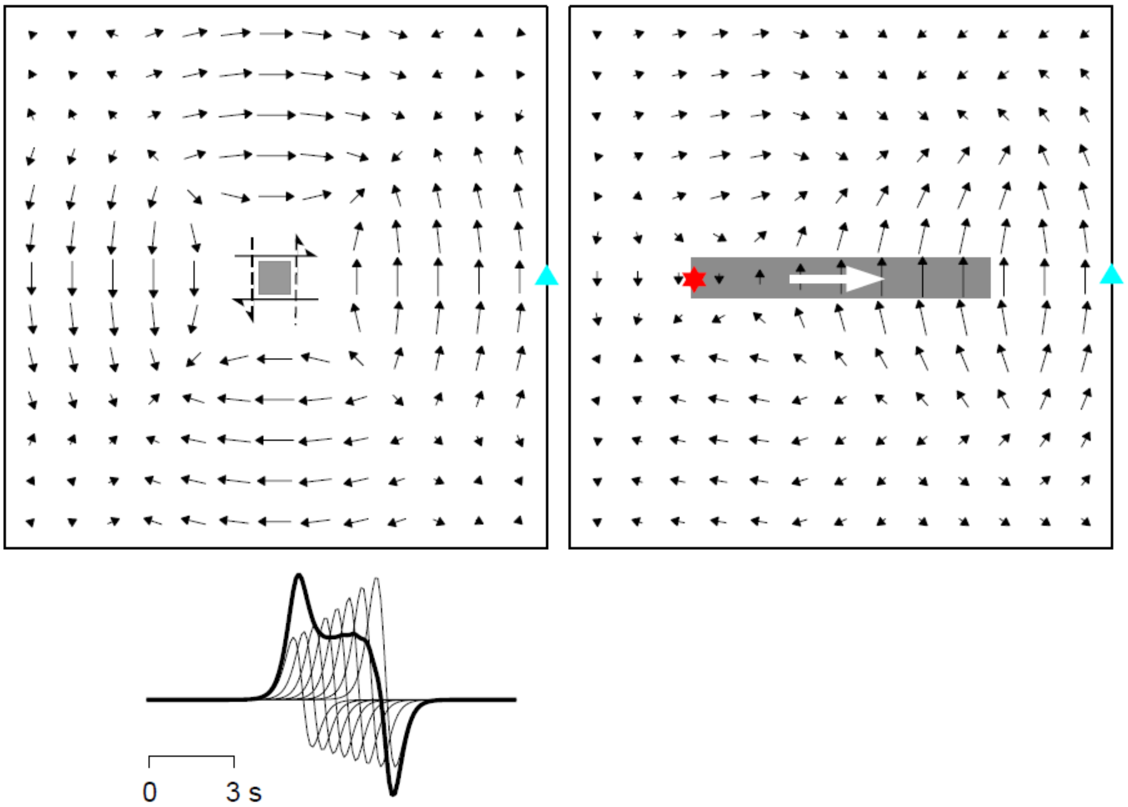

Supplementary Figure 3 | Schematic illustration of rupture directivity for a strike-slip earthquake[4]. Upper left and right diagrams show ground motion patterns of point and finite source models (grey zones), respectively. White arrow indicates rupture direction. Red star is rupture initiation point, and sky blue triangle denotes a station. In lower diagram, thick curve represents constructive interference of ground motions from fault segments (thin curves), illustrating mechanism of rupture directivity pulse from strike-slip finite source.

Supplementary References

1. Takai, N. *et al.* Strong ground motion in the Kathmandu Valley during the 2015 Gorkha, Nepal, Earthquake. *Earth Planets Space*, **68**, doi:10.1186/s40623-016-0383-7 (2016).
2. Galetzka, J., *et al*. Slip pulse and resonance of the Kathmandu basin during the 2015 Gorkha earthquake, Nepal. *Science* **349**, 1091-1095 (2015).
3. Lindsey, E. O. *et al.* Line of sight deformation from ALOS-2 interferometry: Mw 7.8 Gorkha earthquake and Mw 7.3 aftershock, *Geophys. Res. Lett.* **42**, doi:10.1002/2015GL065385 (2015).
4. Koketsu, K. Damaging earthquakes in California and 1995 Kobe earthquake, *Science Journal* **66**, 93-97 (1996).
5. Wessel, P. & Smith, W. H. F. Free software helps map and display data, *EOS Trans. AGU* **72**, 441 (1991).
